# Regulation of phosphorus uptake and utilization: transitioning from current knowledge to practical strategies

**DOI:** 10.1186/s11658-016-0008-y

**Published:** 2016-07-28

**Authors:** Md. Mahmudul Hasan, Md. Mainul Hasan, Jaime A. Teixeira da Silva, Xuexian Li

**Affiliations:** 1 The Key Laboratory of Plant-Soil Interactions, MOE, Center for Resources, Environment and Food Security, Department of Plant Nutrition, China Agricultural University, Beijing, 100193 China; 2 Faculty of Agriculture, Patuakhali Science and Technology University, Dumki, Patuakhali Bangladesh; 3 P. O. Box 7, Miki cho post office, Ikenobe 3011-2, Kagawa-Ken, 761-0799 Japan

**Keywords:** Phosphorus uptake, Phosphorus transporter, Phosphorus use efficiency, Quantitative trait loci, Hormonal signaling, MicroRNA, Arbuscular mycorrhizal fungal symbiosis, Intercropping, Gene overexpression

## Abstract

Phosphorus is a poorly bioavailable macronutrient that is essential for crop growth and yield. Overuse of phosphorus fertilizers results in low phosphorus use efficiency (PUE), has serious environmental consequences and accelerates the depletion of phosphorus mineral reserves. It has become extremely challenging to improve PUE while preserving global food supplies and maintaining environmental sustainability. Molecular and genetic analyses have revealed the primary mechanisms of phosphorus uptake and utilization and their relationships to phosphorus transporters, regulators, root architecture, metabolic adaptations, quantitative trait loci, hormonal signaling and microRNA. The ability to improve PUE requires a transition from this knowledge of molecular mechanisms and plant architecture to practical strategies. These could include: i) the use of arbuscular mycorrhizal fungal symbioses for efficient phosphorus mining and uptake; ii) intercropping with suitable crop species to achieve phosphorus activation and mobilization in the soil; and iii) tissue-specific overexpression of homologous genes with advantageous agronomic properties for higher PUE along with breeding for phosphorus-efficient varieties and introgression of key quantitative trait loci. More effort is required to further dissect the mechanisms controlling phosphorus uptake and utilization within plants and provide new insight into the means to efficiently improve PUE.

## Background

### The need to improve phosphorus use efficiency

Phosphorus is an essential macronutrient for nucleic acid synthesis, membrane buildup and stability, energy metabolism, and many other critical physiological and biological processes during plant growth and development [1, 2]. In spite of the abundance of phosphate in soil [3], phosphorus is poorly available to plants due to its extremely low diffusion rate (10^−12^ to 10^−15^ m^2^/s) [3] and substantial fixation by soil minerals [1, 2].

Phosphorus fixation is the sorption and precipitation of inorganic phosphorus to produce less soluble compounds. In acid soils, H_2_PO_4_^–^ reacts with insoluble oxides of iron, aluminum and manganese. In alkaline soils, soluble H_2_PO_4_^–^ quickly reacts with calcium to form insoluble compounds. This also occurs with the phosphates in fertilizer: when concentrated superphosphate fertilizer is added to soil, the highly soluble mono-calcium phosphate (CaH_2_PO_4_)_4_ reacts rapidly with calcium carbonate (CaCO_3_) to form di-calcium phosphate and again reacts with CaCO_3_ to form tri-calcium phosphate, undergoing further reactions to become even more insoluble (over 1000 times more insoluble than fresh tri-calcium phosphates) [4].

There are many agricultural and biological factors that constrain crop production. Many crops are grown in soils with low phosphorus contents that constrain crop production. Plant root system architecture significantly alters due to environmental stress and nutrient starvation [5, 6]. Plant density, propagule characteristics, weeding regime, environmental stress, ecological niche and plant growth characteristics all play vital roles in determining the yield and nutrient use efficiency of major crops [5–14].

Due to a lack of proper knowledge on plant physiology and molecular biology, farmers tend to use enormous amounts of phosphorus fertilizers during the growing season [15]. Approximately 50 % of applied phosphorus fertilizer is taken up and utilized by plants in a given growing season. The remainder is fixed in soil chemical processes as in the example above or it moves downward into the fresh water system, leading to underground water contamination or eutrophication of aquatic ecosystems [16]. In the worst-case scenario, excess phosphorus lowers the quality of drinking water by promoting the growth of cyanobacteria such as *Microcystis aeruginosa*, which causes toxic algal blooms and produces toxins that can damage the animal liver, intestines and nervous system [15]. In the United States, the annual cost of freshwater eutrophication-related damage is US$2.2 billion, and about half of that damage is due phosphorus deposition [17]. Meanwhile, phosphorus extraction from phosphorite deposits is predicted to plateau in 2035 and geological phosphorus reserves, which are confined to just a few countries, are becoming depleted [18].

It is imperative to understand how plants take up phosphorus and respond to low levels of this nutrient so that effective strategies can be employed to improve phosphorus uptake efficiency (PUpE; the phosphorus concentration inside the plant/the concentration of available phosphorus in the soil) and utilization efficiency (PUtE; the grain weight/total phosphorus in the plant) for higher use efficiency (PUE; combined uptake and utilization efficiency) [19].

## Main text

### Molecular advances in mediating phosphorus uptake and utilization

The concentration of bioavailable H_2_PO_4_^–^ and HPO_4_^2–^ in the soil is generally lower than that in plant tissues (10 μm vs. 5 to 20 mM in general) [3]. Phosphorus is a natural element that is widely distributed with other elements and minerals. Phosphate is a natural compound consisting essentially of salts of phosphorus and other minerals.

Within the plant, most phosphorus is stored in the vacuole. However, phosphate efflux from the vacuole is insufficient to balance the cytosolic phosphate concentration during phosphorus starvation [20]. Under phosphorus-deficient conditions, the cytosolic phosphate concentration decreases rapidly due to insufficient phosphate efflux from the vacuole, which triggers plant adaptive responses to facilitate phosphorus acquisition and translocation [20].

Two primary transport systems mediate phosphorus uptake and transport in plants. The first is an inducible high-affinity transport system with a Km value that generally ranges from 10 to 100 μm/l [21]. It is regulated by the cellular energy supply, intracellular phosphate consumption and the proton electrochemical gradient. The second is a constitutive low-affinity transport system with a Km value in the mM/l range. It transports solutes following the concentration gradient [22].

**Fig. 1:**
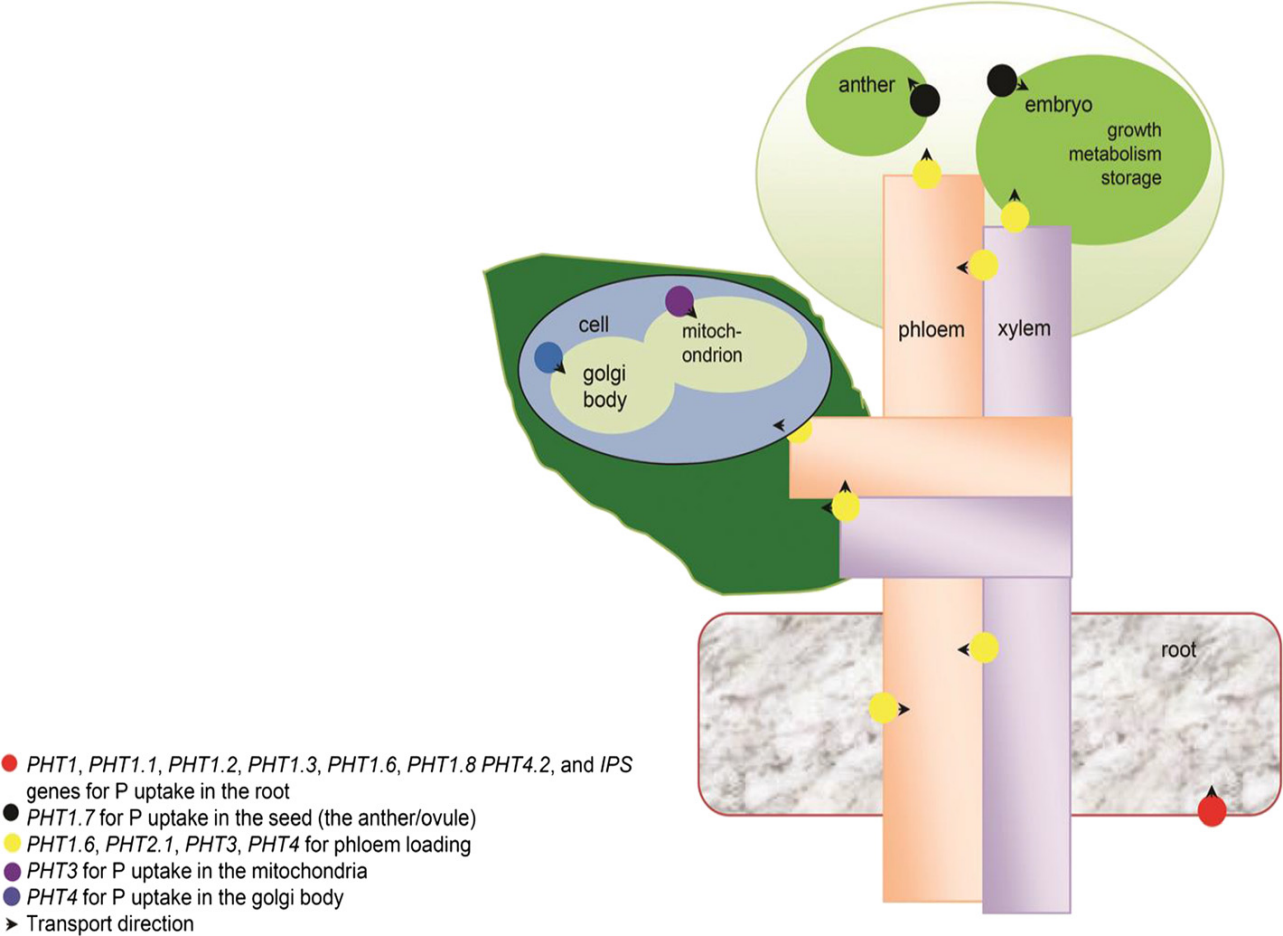
A schematic representation of transporters mediating phosphate transport in plants

There are four families of phosphorus transporters in *Arabidopsis*: PHT1, PHT2, PHT3 and PHT4 (Fig. 1) [23]. The PHT1 family comprises high-affinity phosphate/H^+^ symporters, mostly expressed in the roots [24], with 9, 13, 6 and 8 members in *Arabidopsis*, rice (*Oryza sativa*), maize (*Zea mays*) and barley (*Hordeum vulgare*), respectively [24]. The transcription of *PHT1* transporters is induced by phosphorus starvation. They are responsible for phosphate uptake from the soil and transport to the shoot and the mycorrhizal symbiotic interface [24, 25].

Barley phosphate transporters are the best characterized among all crops [24]. In barley, *PHT1.1* and *PHT1.2* are expressed in root epidermal, cortical and vascular tissues [26]. *HvPHT1.3* expression is detected in the roots [24, 25]. *HvPHT1.3* and *HvPHT1.6* expression correlates with the expression of the non-coding RNA *IPS1* (induced by phosphate starvation 1). The levels of *HvPHT1.3*, *HvPHT1.6* and *IPS1* transcripts increase under conditions of phosphorus starvation [24]. *HvPHT1.6* expression spreads from the root to the shoot [24]. *HvPHT1.7* is expressed in green anthers [24] and *HvPHT1.8* displays a mycorrhiza-specific expression pattern [24, 25]. *PHT1.8* and *PHT1.9* are regulated by ubiquitin E2 conjugase *PHO2*, which controls phosphorus remobilization [24].

In wheat (*Triticum aestivum*; *Ta*), two phosphorus transporters have been characterized thus far: *TaPHT1.2* and *TaPHT1.4* [27, 28]. *TaPHT1.2* is a high affinity phosphorus transporter predominantly expressed in roots, induced under conditions of phosphorus deficiency. TaPHT1 is more commonly expressed in the roots of phosphorus-efficient genotypes (e.g., Xiaoyan 54) than in inefficient ones (e.g., Jing 411) under conditions of phosphorus deficiency and under control conditions [28]. *TaPht1.4* is a high-affinity phosphorus transporter that is specifically expressed in the roots and is highly induced under conditions of phosphorus deficiency. *TaPht1.4* is expressed more during the day than at night. *TaPht1.4* overexpression and knockdown results in improved and sub-optimal growth traits corresponding to more and less phosphorus accumulation, respectively. Therefore, *TaPht1.4* plays a critical role in phosphorus acquisition under conditions of phosphorus deficiency [27]. Knockdown of different phosphorus transporters in the root significantly decreases phosphorus acquisition [27, 29], while overexpression of different phosphorus transporters enhances phosphorus uptake and tolerance of many crops to phosphorus deficiency (Table 1).

**Table 1:** QTLs related to phosphorus uptake, phosphorus use efficiency (PUE) and phosphorus utilization in different crop species

Species	Description	References
*Oryza sativa*	Major QTLs for root length, elongation and internal PUE. Most are located on chromosomes 6 and 9.	[68–70]
*Zea mays*	QTLs for root hair length and plasticity, ear length and diameter, row number/ear, kernel number/row, 100-kernel weight, grain yield/plant for higher PUE. These QTLs are located on chromosomes 1, 3, 4, 5, 9 and 10.	[71, 73]
*Triticum aestivum*	QTLs for tillers/plant, shoot dry weight, shoot and whole plant phosphorus uptake as well as PUE. These QTLs are distributed on chromosomes 1A, 2A, 2D, 3A, 3B, 4A, 4B, 5A, 5B, 5D, 6B, 7A, 7B and 7D.	[72, 107, 108]
*Phaseolus vulgaris*	QTLs for root-hair density, root-hair length, H^+^ exudation and total acid exudation, distributed on different chromosomes.	[74]
*Glycine max*	QTLs for root traits, phosphorus content, total plant biomass and yield traits. These QTLs are mostly located on chromosomes 7, 12 and 17.	[75]
*Brassica oleracea*	QTLs for shoot phosphorus content and PUE, located on chromosomes 3 and 4, respectively.	[77]
*Brassica napus*	QTLs for primary branches, plant height, seed number/pod and pod number/plant, on chromosomes A2, A3 A5 and C6.	[76]
*Brassica rapa*	QTLs for seed phytate, leaf phosphate and phytate contents as well as PUE. The expression QTLs are located on chromosomes A6 and A1, and the phenotypic QTLs are mostly located on chromosomes A1, A3, A8, C1, C3 and C7.	[78]

Within the PHT2 family, *AtPHT2.1*, expressed in chloroplasts, mediates whole-plant phosphate allocation. *Atpht2.1* mutation results in 30 ~ 48 % lower phosphate concentrations in the root and 29–56 % higher phosphate concentrations in the shoot under conditions of phosphorus starvation (Fig. 1) [30]. *AtPHT3* and *AtPHT4* are localized in the mitochondria and Golgi bodies, respectively (Fig. 1) [23]. *PHT4.1* is localized in the thylakoid membrane of the chloroplasts, while *AtPHT4.2*, *AtPHT4.4* and *AtPHT4.5* are expressed in the roots, leaves and leaf phloem tissues, respectively (Fig. 1) [31]. *AtPHT4.6* mediates phosphorus recycling by transporting phosphate out of the Golgi lumen [31].

In addition to phosphorus transporters, genes induced by phosphate starvation (*IPS*) are primarily expressed in the root and shoot vasculature and are involved in regulating phosphorus homeostasis [24]. Overexpression of OsPHR2 (IPS gene; phosphate starvation response 2) and knockdown of OsSPX1 (IPS gene; SYG/PHO81/XPR1 domain 1) synergistically accumulates more shoot inorganic phosphate in rice. In the roots, OsPHR2 upregulates OsPT2 (rice phosphate transporter 2) through physical interaction and upstream regulation of OsPHO2 (PHOSPHATE-RESPONSIVE 2; a phosphate-induced transcription factor and product of MYB DNA-binding domain). OsPT2 is responsible for most of the OsPHR2-mediated accumulation of excess shoot inorganic phosphate. OsSPX1 suppresses OsPT2 by OsPHR2 and the accumulation of excess shoot inorganic phosphate. OsSPX1 is a negative regulator of OsPHR2 and is involved in inorganic phosphate-signaling feedback in roots and depends on OsPHR2 and OsPHO2 [32].

*Arabidopsis* At4 is an IPS gene that is expressed in the vascular tissue under conditions of phosphorus deficiency. The *at4* loss function mutant fails to redistribute phosphorus to the roots correctly in response to phosphorus deficiency and At4 shoots accumulate a greater proportion of phosphorus relative to the wild type. Primary root growth rate in at4 is faster than in the wild type under conditions of phosphorus deficiency [33].

There are five IPS genes in *Arabidopsis* and two in monocot (rice, maize, barley) genomes [24]. *AtIPS1* overexpression reduces shoot phosphorus accumulation [34]. *PHO2* participates in phosphorus signaling by regulating *PHT* and *PHO1* expression [26]. *AtPHO2*, which is a ubiquitin E2 conjugase, modulates phosphorus deficiency-dependent miR399-mediated transcript decay. In the *Atphr1* mutant, wider ranges of inorganic phosphate-responsive genes are altered and expression of miR399 is enhanced. Therefore, the miR399/APHO2 pathway is a subcomponent of the inorganic phosphate-signaling network, operates downstream of AtPHR1 and regulates phosphorus allocation between the shoot and the root. *AtPHO2* has close homologs in crop plants and phosphorus-dependent miR399 expression is conserved, as observed in rice (*Oryza sativa*) [26].

WRKY75 is one of several transcription factors induced during inorganic phosphate deprivation. RNAi-mediated silencing of WRKY75 resulted in early anthocyanin accumulation, indicating that RNAi plants are more susceptible to phosphorus stress. WRKY75-RNAi suppresses several phosphorus deficiency-induced genes, including Mt4/TPS-like genes and those for phosphatases and high-affinity inorganic phosphate transporters, thus decreasing phosphorus uptake. Independent of phosphorus availability, WRKY75-RNAi results in increased lateral root length and number and root hair number. However, changes in the root architecture were obvious under both inorganic phosphate-sufficient and -deficient conditions. WRKY75 is the first member of the WRKY transcription factor family involved in phosphorus acquisition and root architecture changes [35]. AtZAT6 (zinc finger of Arabidopsis 6), a cysteine-2/histidine-2 zinc finger transcription factor, is induced during phosphorus deficiency. ZAT6 overexpression decreases phosphorus acquisition, affects root development, alters root architecture and retards seedling growth. ZAT6 regulates root development independently of phosphorus levels and influences phosphorus acquisition and homeostasis [36].

Roots respond to a variety of environmental stimuli with high plasticity. In cases of insufficient phosphorus supply, plants may sense external signals and stimulate root growth to increase overall uptake capacity. In *Arabidopsis*, low phosphorus suppresses primary root growth with enhanced lateral root and root hair growth, as well as higher density of lateral roots, which definitely promotes compensatory phosphorus uptake [37, 38]. Low phosphorus inhibits cell division in the primary root meristem and promotes differentiation processes within the root tip, in which the quiescent center can sense low phosphorus signals [39]. Low phosphorus generally leads to a higher root-to-shoot ratio in maize [40]. Furthermore, after six days of phosphorus starvation, the growth of primary maize roots is slightly promoted, with a significantly reduced number of lateral roots and lateral root primordia.

The results of a transcriptomic analysis of the maize lateral root primordium revealed that auxin signaling participates in root morphology modification under conditions of low phosphate availability, probably via changes in local auxin concentration, biosynthesis and transport of auxin and LOB (lateral organ boundary) domain proteins [40].

It has also been found that approximately 22-nt miRNAs generated by double-stranded RNA degradation play important biological roles in regulating a wide array of genetic, epigenetic and developmental processes, as well as responses to environmental stresses [41]. Table 2 lists these in detail. miR399, the first characterized microRNA, functions as a systemic signaling molecule coordinating whole-plant phosphate homeostasis [26]. Under conditions of phosphorus starvation, miR399 accumulates in the phloem, regulating phosphorus uptake and allocation through three target genes in a conserved manner: *PHT1.7*, *PHO2* and a dead box helicase in *Arabidopsis* [42–44]. miR399 guides *PHO2* mRNA degradation to regulate phosphorus homeostasis [26, 45]. *AtPHO2* mRNA degradation is inhibited when an RNA duplex with a mismatch loop forms between *AtIPS1* and miR399 due to incomplete sequence complementarities [46]. The miR399–*PHO2* pathway is further regulated by *PSIs* (phosphorus starvation-induced genes) [38, 47]. miR395 downregulation stimulates expression of its target genes *APS4* and *SULTR2.1*, probably facilitating sulfate assimilation, translocation and sulfolipid biosynthesis [48]. Other common phosphorus starvation-responsive miRNAs include miR156, miR159, miR166, miR319, miR395, miR398 (the complementary strand of miR2111), miR447 and miR827. miR156, miR778, miR827 and miR2111 are induced under conditions of phosphorus starvation, while miR169, miR395 and miR398 are repressed [43, 48, 49]. Many miRNAs have been identified in cereal crops (maize, wheat, and barley) under conditions of phosphorus starvation [49–51]. In white lupin, 35 miRNA families are differentially expressed in the root, stem and leaf tissue under conditions of phosphorus deficiency [52]. Similarly, miRNAs (including novel miRNAs) are differentially expressed in the root and shoot in soybean (*Glycine max* L.), tomato (*Solanum lycopersicum* L.) and rapeseed (*Brassica napus*) in response to phosphorus starvation [43, 53, 54].

**Table 2:** Major miRNAs involved in the response to phosphorus starvation in different crop species

Species	miRNA	Description	References
Common in many species	miR399	A systemic signaling molecule mediating whole-plant phosphorus homeostasis	[26, 32, 42, 43, 45]
Common in many species	miR156, miR159, miR166, miR319, miR398, miR447, miR827, miR156, miR778, miR827, and miR2111	Induced by phosphorus starvation	[43, 48, 49]
Common in many species	miR169, miR395 and miR398 are repressed.	Repressed by phosphorus starvation	[43, 48, 49]
*Zea mays*	miRNA399b and miR3	Induced by *ZmPT1* and *ZmPT2* by phosphorus starvation	[48]
*Triticum aestivum*	mir159b, mir167, mir399, mir408, mir1122, mir1125, mir1135 and mir1136	Induced by phosphorus starvation	[51]
*Brassica napus*	miR169, miR827 and miR2111	Responsive to phosphorus starvation	[44]
*Solanum lycopersicum*	miR319 and miR394	Expressed in roots and induced by phosphorus starvation	[54]
*Solanum lycopersicum*	miR158, miR169g, miR172, miR172b, miR319, miR771 and miR775	Expressed in leaves and repressed by phosphorus starvation	[54]
*Lupinus albus*	35 miRNA families	Phosphorus starvation upregulates 17, 9, 10 miRNAs in the root, stem and leaf, respectively, and represses 7, 6, 12 miRNAs in the root, stem and leaf, respectively	[52]
*Hordeum vulgare*	55 potential sRNAs	Regulated by phosphorus starvation and responsible for phosphorus homeostasis	[50]
*Glycine max*	110 novel miRNAs	Responsive to phosphorus starvation, with 55 expressed in the root and 55 in the shoot	[53]

In addition to miRNAs, some 500- to 700-bp non-coding RNAs (*IPS1* and *At4* in *Arabidopsis*) are highly induced by phosphorus starvation [33]. The *At4* loss-of-function mutant and *AT4/PHT1.1/PHT1.4* triple mutant respectively show 17 % and 49 % higher shoot phosphorus content. *At4* overexpression causes less shoot phosphorus accumulation [33, 34]. With more and more miRNAs characterized, their vital functions and molecular pathways in controlling the plant response to low phosphorus are becoming increasingly clear, solid and applicable. Using artificial miRNA, scientists are able to modulate phosphorus uptake and metabolic processes to enhance PUE, crop growth and yield formation under conditions of low phosphorus availability using reliable transgenic technologies [55].

As observed above, phosphorus deprivation has diverse effects on root growth, elongation and biomass accumulation, probably due to the different genotypes, and the levels and duration of phosphorus starvation. Low phosphorus also influences the root architecture, including primary root length, lateral root initiation and root hair density [38–40].

With regard to phosphorus transporters, corresponding regulation and root architectural responses to phosphorus starvation, several essential concerns remain elusive. How are they genetically regulated? What are their temporal dynamics under conditions of low phosphorus availability? How do they contribute to crop tolerance to phosphorus starvation in the field? Do they have breeding values in a given crop species? To address these questions, much more effort is needed to investigate the underlying genetic, developmental and physiological mechanisms of phosphorus uptake and utilization in major crops.

### Metabolic changes to plants facing phosphorus deficiency

Plants undergo a series of metabolic alterations to maintain cytoplasmic phosphate concentrations and intracellular ATP and nucleotide levels upon phosphorus starvation. Among these adaptations, phosphorus starvation-induced intracellular acid phosphatases are involved in phosphate remobilization and recycling from intracellular phosphate reserves. Phosphorus deficiency induces de novo synthesis of extra- and intracellular *Arabidopsis* purple acid phosphatase. Phosphorus starvation causes the over-accumulation of anthocyanin due to the higher expression of F3’H (flavone 3’ hydroxylase), leucoanthocyanidin dioxygenase, PAP1 (for production of anthocyanin pigment 1, AtMYB75) [56], and UDP-Glc-flavonoid 3-*O*-glucosyltransferase transcripts. It inhibits crop growth and development and significantly reduces economic yield [57].

AtPAP12 and AtPAP26 scavenge phosphorus from extracellular phosphorus-esters, whereas the dual-targeted AtPAP26 functions in vacuolar inorganic phosphate recycling under conditions of phosphorus deficiency, when purple acid phosphatase (PAP) isozymes are likely upregulated and could be engineered to develop a phosphorus-efficient crop [58]. Phosphorus deficiency-mediated regulation of PAPs at transcriptional and post-translational levels has been characterized in *Arabidopsis*, rice and maize [58]. PAP15 plays a dominant role in phosphorus remobilization from phytate reserves. *AtPAP15* overexpression in carrot (*Daucus carota*) significantly improves plant growth and phosphorus use efficiency (PUE). Overexpression of AtPAP15 containing a carrot extracellular targeting peptide showed a 1.5-fold increase in Apase activity in soybean hairy roots. Such plants showed increased phosphorus content and dry weight by as much as 90.1 % and 117.8 %, respectively, compared to non-transformed controls grown on sand culture containing phytate as the sole phosphorus source. Overexpressing soybean (*Glycine max*) shows significantly higher APase and phytase activity in leaf and root exudates, respectively. The plants showed increased pod numbers per plant and seed numbers per plant when grown on soil (59.0 % and 66.7 %, respectively) [59].

A β-conglycinin promoter (α’-subunit) mediates the seed-specific expression of the soybean phytase gene. Its action alters soybean seed phytate content during seed development and degrades phytic acid (IP6) accumulation. *GmPhy* gene-expressing soybean seeds showed decreased IP6 levels compared to the control, and ectopic phytase expression during seed development reduced phytate content in soybean seeds, which therefore increased phosphorus availability [60]. Phytate (inositol hexakisphosphate, IP6) is a regulator of intracellular signaling and phosphate storage in plant seeds. Coincident disruption of inositol polyphosphate kinases, AtIPK1 and AtIPK2, gave rise to normal seed yield with nearly ablated seed phytate and no accumulation of phytate precursors. It also increased seed-free phosphate 10-fold. Inositol tetrakisphosphate (IP4) and inositol pen-takisphosphate (IP5) 2-kinase activity are required in phosphate sensing and root hair elongation.

Phytate, or inositol hexakisphosphate (IP6), is responsible for approximately two-thirds of total seed phosphorus, and comprises 1 % of the total dry weight [61]. In mature desiccated seeds, IP6 comprises 100 % of the detectable IPs, comprises an average of 22 nmol IP6 mg seed, 1.4 % by weight. In the *atipk1* mutant and *atipk1-1 atipk2-1* double mutant, seed phytate levels are reduced by 35–83 % and 95 %, respectively. Grain phytate content is inversely related to inorganic phosphate levels. The *atipk1-1* and *atipk2-1* mutants showed ~2-fold increase in inorganic phosphate levels, and the double mutant showed a 10-fold increase. The *atipk1-1* mutant showed altered phosphate homeostasis and suffers from phosphate toxicity under optimum phosphate conditions. To scavenge more inorganic phosphate from the rhizosphere under phosphate starvation conditions, *Arabidopsis* seedlings produce longer root hairs [62]. Irrespective of inorganic phosphate status, the root hairs of the *atipk1-1* mutant were higher in number than those of the wild-type plant.

Enhanced survival is largely a function of stress-tolerant mechanisms rather than a net improvement in plant production under severe stress, but such conditions will most probably not enhance plant yield in moderate stress. Under mild stress, plants maintaining more growth and photosynthesis could open a new exciting paradigm for trait identification [63, 64].

Several PAPs have acid phosphatase and alkaline peroxidase activities, and their overexpression increases phosphorus starvation tolerance [59] by protecting plants from oxidative damage [58]. At the subcellular level, alterations in mitochondrial electron transport, cytosolic glycolysis and tonoplast H^+^ pumping facilitate respiration and vacuolar pH maintenance with insufficient phosphorus supply.

During phosphorus deficiency, roots could probably excrete excess organic acids produced by PEPC, malate dehydrogenase (MDH) and citrate synthase (CS) to increase mineral-bound phosphorus (by solubilizing calcium, iron and aluminum phosphates = Met-Pi), organic phosphorus and hydrolysis by excess PAPs. Under such conditions, vacuolar PAPs are upregulated and recycle phosphorus from monoesters. Electron transport utilizes energy conservation metabolic pathways in the mitochondria and the citric acid cycle consumes less ATP [61, 62]. Phosphorus starvation leads to upregulated transcription of glycolytic bypass enzymes – specifically ATP-dependent phosphofructokinase (PFK), generating fructose 1,6-biphosphate; NADP-dependent glyceraldehyde-3phosphorus dehydrogenase (NADP-G3PDH) that bypasses inorganic phosphate-dependent NAD-G3PDH and phosphoglycerate kinase; and the PEPC, MDH and NAD-malic enzymes that can bypass pyruvate kinase (PK) – and the inorganic pyrophosphate H^+^-pump (H^+^-PPiase) [63].

Using bypass enzymes, plants avoid ATP-limited reactions, and take advantage of their limited ATP reserves and PPi-dependent phosphate recycling to overcome phosphorus starvation. Overexpression of tonoplast H^+^-PPiase results in higher rates of plant growth under conditions of phosphorus starvation due to rhizospheric acidification [65]. The cluster roots exude 20- to 40-fold more organic acid (mainly citrate and malate) resulting in the exudation of about 10–25 % of the total plant carbon. Low phosphorus-induced internal organic acid is not proportional to the quantity of released exudates. Hence, synthesis of exudates (mainly malate and citrate) under conditions of phosphorus deficiency is specific and selective. CO_2_ fixation and pulse-chase labeling measurements with photosynthetic CO_2_ fixation provide about 65 % of the exuded carbon, while dark CO_2_ fixation in cluster roots provides about 35 %. CO_2_ fixation in cluster roots is increased by enhanced activity of phosphoenolpyruvate carboxylase (PEPC), malate dehydrogenase (MDH) and citrate synthase (CS) in white lupin (*Lupinus albus* L.), chickpea (*Cicer arietinum* L.), rapeseed (*Brassica napus* L.), and *Sesbania rostrata.* Aconitase (AC) activity and respiration also reduce in a phosphorus-deficient cluster. Therefore, a coordinated induction at the molecular level is involved in developmental and biochemical changes [63].

Beyond internal metabolic changes, plants exude organic acids into the rhizosphere to immobilize chemically trapped phosphorus, increase organic phosphorus solubility and promote symbiotic microbial growth for more phosphorus uptake [63]. The findings of our most recent studies of white lupin revealed that higher light intensity (600 μmol/m^2^/s vs. 200 μmol/m^2^/s) increases the number of cluster roots per plant (~140 vs. ~25), number of lateral roots per plant (~150 vs. ~100), total root length (~2500 vs. ~1000 cm/plant) and root surface area (~300 vs. ~100 cm^2^/plant) under phosphorus-deficient (1 μM) conditions, suggesting that phosphorus use efficiency is directly related to carbon metabolism [66].

A better understanding of metabolic adaptations of plants to long-term low phosphorus levels is still needed. Recently developed approaches for metabolome analysis should be applied together with transcriptomic profiling for comprehensive assessments of the metabolic dynamics of staple crops coping with insufficient phosphorus supply. Functional dissection of key genes and gene networks could then be performed to identify candidate genes for molecular breeding or combinatory gene overexpression for immediate agronomic trait improvement.

### QTLs related to phosphorus uptake and utilization under conditions of low phosphorus availability

Yield is determined by many agronomic traits. These traits are correlated by QTLs [67]. *Pup1* is a well-characterized QTL that encodes a protein kinase stimulating early root growth. Absent in the rice reference genome and other phosphorus-sensitive modern varieties, *Pup1* was found only in a traditional aus-type rice variety grown in low phosphorus areas in northeast India. Table 1 details some of the findings. Introgression of *Pup1* into phosphorus-sensitive varieties or *Pup1* overexpression significantly enhances root growth and grain yield under conditions of phosphorus starvation, which demonstrates the remarkable breeding value of *Pup1* in low phosphorus-tolerant rice development [68, 69]. More QTLs are detected in relation to root elongation, phosphorus uptake, vegetative and reproductive growth, plant height, tiller number, 1000-grain weight and dry weight under conditions of low phosphorus availability [70]. Other QTLs related to phosphorus uptake and utilization have been identified in maize and wheat under conditions of phosphorus starvation [71–73]. For common bean, a number of known QTLs are related to root development and phosphorus acquisition and accumulation [74]. QTLs play roles in mediating plant growth and phosphorus uptake and PUE in response to low phosphorus in soybean [75]. Similar QTLs have also been detected in *B. napus*, *B. rapa* and *B. oleracea* under conditions of phosphorus starvation [76–78]. Although a large number of QTLs are involved in responses to low phosphorus in terms of root and/or shoot growth, phosphorus uptake and utilization, and grain development and yield formation in a variety of crop species, few have been precisely mapped and characterized with regard to their biological functions. Substantial genetic and molecular work is required for fine QTL mapping and functional investigation. This will shed more light on how PUE is genetically controlled by complex genetic networks and lay a solid basis for maker assisted phosphorus-efficient crop breeding.

### Involvement of hormone signaling in shaping root architecture with insufficient phosphorus supply

Hormones (ethylene, auxin and gibberellins) play essential roles in regulating responses to low phosphorus levels and phosphorus signaling. One of these essential regulations is that these hormones mediate root responses to low phosphorus levels. Ethylene restricts primary root growth, alters the root growth angle and stimulates root hair development, similar to root phenotypes of *Arabidopsis* under conditions of low phosphorus availability [79]. Ethylene restricts primary root growth in response to low phosphorus levels by modulating cell division in the quiescent center in *Arabidopsis* [80] Auxin is another essential player mediating root architecture under conditions of low phosphorus availability. Exogenous auxin affects root architecture in the same way that low phosphorus levels do [38, 81]. Auxin regulates root growth under conditions of low phosphorus availability via auxin redistribution. Triiodobenzoic acid (TIBA) inhibits primary root elongation in *Arabidopsis* under conditions of low phosphorus availability due to higher auxin concentrations in the root meristem and increased auxin sensitivity [81].

In addition to ethylene and indole-3-acetic acid (IAA), gibberellins (GAs) are also critical regulators in the plant response to phosphorus deficiency. Gibberellic acid (GA_3_) promotes plant growth due to imposed growth restraint by a family of DELLA proteins. DELLA proteins comprise five distinct proteins that are subsequently polyubiquitinated by the SCF SLY1/SLY2 E3 ubiquitin ligase to target 26S proteasome destruction. DELLAs restrain plant growth, whereas GA stimulates by destroying DELLAs. The GA–DELLA signaling pathway controls plant phenotypes during inorganic phosphate starvation. DELLA-deficient mutants do not exhibit inorganic phosphate starvation growth responses without alteration of GA sensitivity of 26S proteasome-dependent DELLA destruction. Therefore, inorganic phosphate starvation causes DELLAs to accumulate by reducing bioactive GA. It also suppresses root growth of *Arabidopsis* [57, 82]. Exogenous GA can restore root growth under conditions of low phosphorus availability [83]. Other hormones such as jasmonic acid and strigolactones may also be involved in regulating root growth and development, and many other low phosphorus responsive pathways in *Arabidopsis* [84, 85].

## Conclusions

### Strategies to improve phosphorus use efficiency

#### Arbuscular mycorrhizal fungal symbiosis and its application for higher phosphorus use efficiency

Most terrestrial plant species (70–80 %) can interact with arbuscular mycorrhizal fungi (AMF), which are mycorrhiza that penetrate the cortical cells of the roots of vascular plants, and ultimately help in efficient phosphorus uptake [25]. Following invasion into the plant root, the fungus enters inner cortex and develops highly branched hyphae. Arbuscules then develop at the ends of the hyphae and are enveloped by the root cell plasma membrane, creating an efficient signaling and nutrient exchange interface between two organisms. Fungal hyphae extensively spread outside the roots, easily penetrating narrow soil pores to retrieve phosphorus nutrients [86]. The fungi obtain carbon from the host and release phosphate to the periarbuscular interface for host uptake via phosphorus transporters in the cortical cells [25]. In AMF symbiotic plants, phosphorus is absorbed through direct and mycorrhizal uptake pathways [25]. The contribution of the mycorrhizal pathway to the total phosphorus uptake is highest when the phosphorus available in the soil is at the optimal level for AMF infection and symbiosis. Low or high levels of soil-available phosphorus reduce the contribution of the mycorrhizal pathway to total phosphorus uptake (Qun Chu, China Agricultural University, unpublished data).

Phosphorus starvation and corresponding signaling of the host plant promote infection and AMF symbiosis [25]. Rice takes up more than 70 % phosphorus via AMF in a symbiotic system [87]. The efficiency of AMF symbiosis and phosphorus translocation declines with increasing phosphorus levels in the soil [28].

OsPHT1.11 is the first AM-specific phosphorus transporter in rice and its homologous mutation (MEDtr; PHT1.4) blocks symbiotic phosphorus uptake in the periarbuscular interface of *Medicago truncatula* [88]. OsPHT1.11 is also essential for AMF symbiosis in rice. Its homologous proteins in monocots and dicots show high levels of similarity with ancient moss counterparts, indicating its ancient origin. Both *OsPHT1.11* and *OsPHT1.13* are vital in an AMF symbiosis, suggesting that grasses probably employ a specialized strategy for phosphorus acquisition through symbiosis with AMF [89]. Phosphorus transporters of AMF mediate phosphorus uptake and transporter expression levels of the host plant in a symbiotic system. AMF PHT1 mediates phosphorus uptake at the root periphery, and its knockdown causes phosphorus deficiency in the host plant [23, 90]. AMF infection increases *ZmPHT1.3* expression 44-fold and decreases *ZmPHT1.6* expression 135-fold in maize compared to the non-infected control [90]. AMF colonization also influences auxin and ethylene levels that regulate lateral root and root hair development in the host plant [91].

Symbiosis with fungi enables the host plant to obtain large amount of phosphorus nutrients. Tomato *PHT1.4* (*PT1.11* homolog in tomato) affects neither symbiosis nor mycorrhizal phosphorus uptake [26]. Direct phosphorus uptake dominates in dicots (e.g., *Linum usitatissimum*, tomato and *M. truncatula*) [25].

Mycorrhizal inocula can be depleted due to locally excessive phosphorus fertilization, long fallow periods, and non-host crop cultivation. In agricultural systems, it is necessary to inoculate cereal crops with proper AMF, which not only takes advantage of the biopotential for efficient phosphorus mining and acquisition, but also reduces phosphorus fertilizer consumption and environmental pollution.

Regardless of phosphorus levels, inoculation of maize plants with AMF dramatically increases phosphorus uptake and grain yield [92]. It is also important to select crop species-preferred AMF and strains that infect efficiently, consume less carbon, and provide more phosphorus to the host.

#### Intercropping for higher phosphorus use efficiency

Intercropping is a very common agronomic practice in many countries in Asia, Africa and South America, primarily because it provides a higher land equivalent ratio and beneficial interspecies interactions [93, 94]. Maize–fava bean (*Vicia faba* L.) intercropping promotes phosphorus uptake and yield formation owing to interspecific mutualisms for phosphorus mobilization and the release of acid phosphatase, protons and/or carboxylates in the rhizosphere that increase the soluble inorganic phosphorus concentration [94]. Maize genotypes with larger roots have even larger stimulatory effects on phosphorus uptake and accumulation during phosphorus starvation. In the maize–chickpea intercropping system, chickpea helps organic phytate mobilization and facilitates organic phosphorus utilization by exuding acid phytase. Compared to other crops, legumes exude more organic acids to increase phosphorus solubility in the rhizosphere [95]. Picidic acid exuded by the pigeon pea roots promotes phosphorus release from FePO_4_ through iron chelating, and citric and malic acids from lupine (*Lupinus arizonicus* L.) root exudates facilitate phosphorus uptake under conditions of phosphorus starvation [95, 96].

Thus, crops may exude i) organic acids or acid phytase to activate chemically fixed inorganic and organic phosphorus in the soil; ii) carboxylates to chelate Al, Ca, Fe and release phosphate bound to these metal ions; or iii) phosphatase to decompose organic phosphorus [95, 96]. Therefore, intercropping with appropriate crop species is an important agronomic practice to improve PUE in soils with limited phosphorus.

#### Tissue-specific homologous gene overexpression for improving phosphorus use efficiency

In spite of substantial molecular advances, the large and complex crop genomes make gene functional studies an extremely challenging task. Time-consuming and laborious forward genetic analysis also hampers large-scale functional investigation of crop genes and QTLs involved in phosphorus uptake, translocation and utilization [97]. Consequently, it remains largely unclear how crops respond to low phosphorus conditions in terms of genetic regulatory pathways.

The traditional breeding process takes a long time, and phenotypic selection for optimized root systems under various environmental conditions is extremely challenging. Current molecular technologies have greatly facilitated crop breeding or introgression of beneficial agronomic traits. However, molecular marker-assisted breeding generally takes 7–10 years, and the achievements of molecular marker-assisted selection for low phosphorus tolerant varieties are very limited [98, 99]. Given the essential nature and potential conservation of phosphorus uptake and metabolism across species, homologous gene overexpression may be a powerful approach to investigate molecular regulatory mechanisms in crops under conditions of low phosphorus availability and develop phosphorus stress-tolerant varieties, with less time and a larger probability of success than traditional breeding [100]. Indeed, transporter overexpression has been shown to improve nutrient use efficiency [101]. To date, a variety of gene transformation techniques have been developed for many crop species [97, 101–103] and proper gene overexpression enhances phosphorus acquisition and content, suggesting that appropriate gene overexpression in different crops not only promotes phosphorus acquisition, but also sheds light on regulatory and signaling mechanisms of phosphorus uptake in crops (Table 3). Homologous gene overexpression for these transporters (Tables 3 and 4), transcription factors, and regulators in phosphorus signaling and regulatory pathways may provide novel insights into their biological functions and have previously unpredicted potential to increase PUE (Fig. 1, Tables 3 and 4).

**Table 3:** Genes overexpressed in different crops for higher phosphorus use efficiency

Gene	Major functions	References
Proton-pyrophosphatase gene *AVP1/AVP1D* (*AVP1DOX*)	Enhances phosphorus acquisition, root branching and overall mass in *Arabidopsis*, rice, and tomato	[109–111]
*GmACP1*	Overexpression in soybean hairy roots increase phosphorus efficiency by 11–20 %	[112]
*ZmPTF1*	Improves low phosphorus tolerance in maize by regulating carbon metabolism and root growth	[104]
*OsPTF1*	Results in a 30 % increase in the phosphorus content in transgenic plants	[100]
*OsPht1-8*	Involved in phosphorus allocation and remobilization	[29]
*AtPHR1*, *OsPHR1*, *OsPHR2*	Enhance the expression of different phosphorus transporters and increases shoot phosphorus concentration	[105, 106, 113]
*OsPT1*, *OsPT6*	Increase phosphorus acquisition and shoot phosphorus accumulation	[114, 115]
*LaGPX-PDE1*, *LaGPX-PDE2*	Regulate root hair development and enhance glycerophosphodiester turnover	[116]
*AtPHO1*, *AtPHO1-H1*, *AtPHO1-H10*	Increase phosphorus acquisition during phosphorus starvation	[117]
*GmEXPB2*	Increases phosphorus acquisition by enhancing lateral root elongation	[118]
*AtPAP15*	Enhances intracellular APase activity in the leaves and increases phosphorus use efficiency and yield	[59]
*PvPS2-2*	Increases phosphorus acquisition efficiency by increasing root dry weight, root hair numbers and total phosphorus content in root hairs	[119]
*LASAP2*, *OsPAP10a* (NC_008394.4)	Increase dry matter production and phosphorus accumulation under phosphorus-deficient conditions	[120]
*TIR1*	Stimulates lateral root formation upon phosphorus starvation	[121]
*AtAVP1OX*, *LeAVP1D-1*, *LeAVP1D-2*	Increase the density of root hairs during phosphorus starvation	[65]
*At2g01060*	Increases shoot phosphorus accumulation irrespective of phosphorus supplies	[122]

**Table 4:** Candidate genes for overexpression to increase phosphorus use efficiency

Gene	Response to phosphorus starvation	References
*Cm-PAP10.1*, *Cm-PAP10.2*, *Cm-RNS1*	Highly upregulated upon phosphorus starvation, with potential to increase phosphorus remobilization efficiency	[123]
*AtLPR*	Upregulated under conditions of phosphorus starvation, promoting lateral root formation and phosphorus use efficiency via down-regulating auxin suppressors	[121]
*AtPAP26*	Confers *PSI* intracellular (vacuolar) APase activity	[124]
*TaALMT1*	Increases phosphorus acquisition efficiency and grain yield	[125]

Notably, tissue-specific overexpression seems a promising strategy to reduce public concerns regarding transgenic foods. With the improvement of crop transformation protocols and establishment of reliable transformation and mutant screen facilities, homologous gene overexpression in crops may be a practical and plausible way to improve PUE.

#### Challenges and future work

**Fig. 2:**
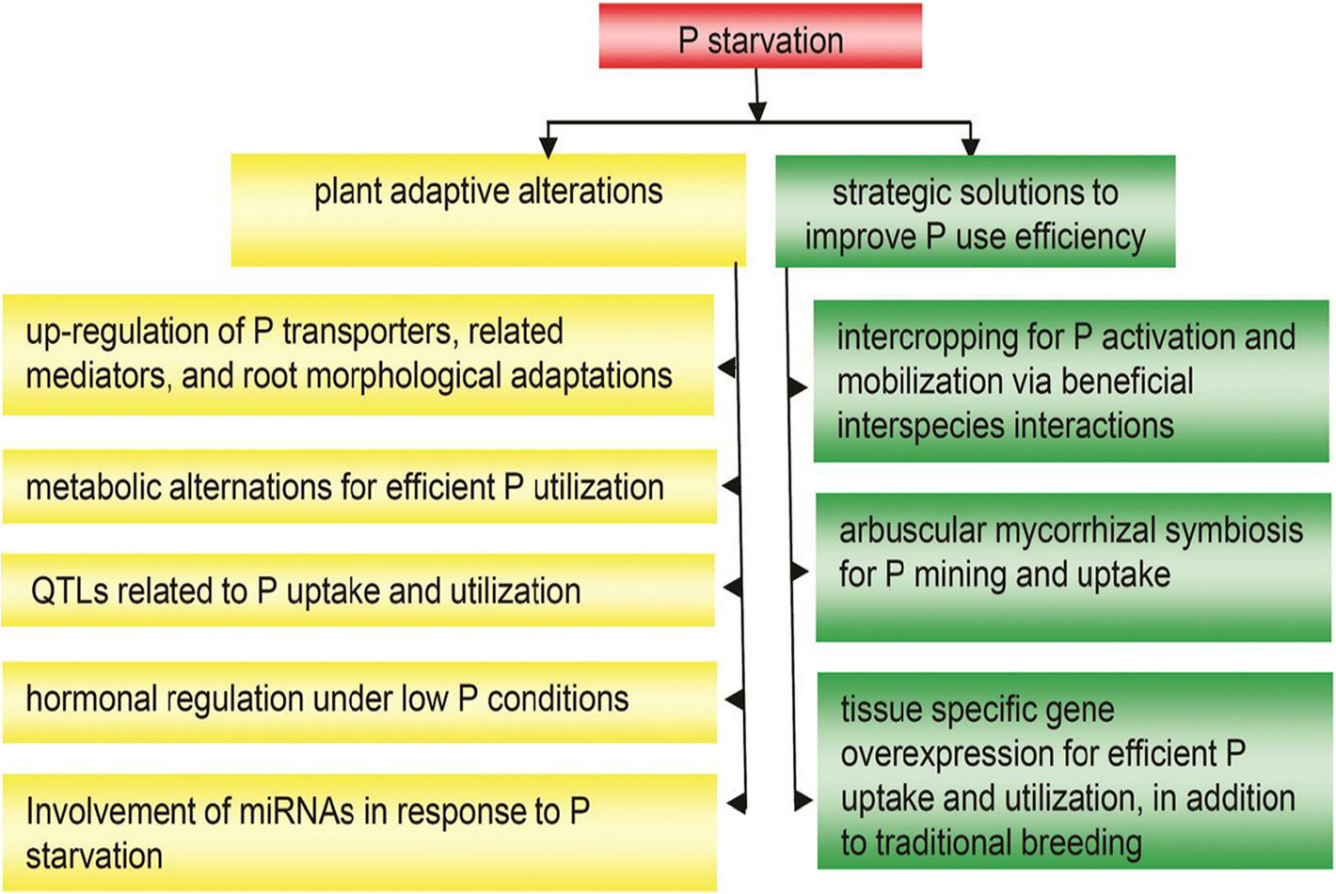
An overview of plant responses to phosphorus starvation and strategic solutions to improve phosphorus use efficiency

Despite numerous reports on the different responses of plants respond to varying phosphorus supplies (Fig. 2), systemic and detailed mechanisms have yet to be elucidated. More research is needed in six areas:

i) Transporter characterization in major crops;
ii) Large-scale metabolome profiling with regard to phosphorus;
iii) Fine QTL mapping to reveal complex genetic pathways controlling critical agronomic traits;
iv) Integration of information on regulatory networks with hormone signaling and microRNAs;
v) Study of the underlying molecular mechanisms of intercropping facilitation; and
vi) Investigation of phosphorus uptake and translocation via symbiotic AMF.

Although strategic solutions to improve PUE have been made, a thorough mechanistic dissection of these issues is essential. While transgenic plants are valuable resources for functional characterization of the transformed gene, homologous gene overexpression may fail to enhance PUE due to species variation. False positive results may also occur due to the overdose of a candidate gene. Clean knockout or knockdown mutant lines should be used to resolve this issue.

## Abbreviations

AMF: Arbuscular mycorrhizal fungal
PUE: Phosphorus use efficiency
QTL: Quantitative trait loci
